# Assessment of Screening, Brief Intervention, and Referral to Treatment Training to Interprofessional Health-Care Students

**DOI:** 10.1177/2377960819834132

**Published:** 2019-04-29

**Authors:** Helen C. Pervanas, Eric Landry, Douglas R. Southard, Pamela P. DiNapoli, Paula Smith, Jennifer Towle, Kate Semple Barta, Kristina Fjeld-Sparks, Devona Stalnaker-Shofner

**Affiliations:** 1 MCPHS University, Manchester, NH, USA; 2 CVS Health, West Lebanon, NH, USA; 3 Franklin Pierce University, West Lebanon, NH, USA; 4 University of New Hampshire, Durham, NH, USA; 5 Southern New Hampshire Area Health Education Center, Raymond, NH, USA; 6 The Dartmouth Institute for Health Policy and Clinical Practice, Hanover, NH, USA; 7 Antioch University New England, Keene, NH, USA

**Keywords:** substance misuse, screening, interprofessional education, screening, brief intervention, and referral to treatment

## Abstract

Substance abuse and addiction are responsible for an assortment of health and financial concerns in the United States. Tools to identify and assist at-risk persons before they develop a substance use disorder are necessary. Screening, brief intervention, and referral to treatment (SBIRT) can be utilized by health-care professionals to identify those at risk to minimize health-related complications and the potential of developing a substance use disorder. The primary objective of this study was to provide educational training sessions on SBIRT to health-care students utilizing interprofessional education activities and assess perceptions of the training sessions and activities with regard to confidence to utilize SBIRT in at-risk patients and overall student satisfaction with SBIRT instruction. The research protocol enrolled students of pharmacy, nursing, medicine, behavioral health, and physician assistant studies who received interprofessional SBIRT training. Students completed an anonymous posttraining online survey, measuring student perceptions of knowledge gained and confidence to utilize training. A total of 303 students completed the SBIRT training. Approximately 70% of students were satisfied with the training materials, instruction, quality, and experience. After training, 78% were confident that they could perform screening for substance abuse, conduct a brief intervention (80%), and when to refer to treatment (71%). A total 73% of students reported that the asynchronous online-based activity was extremely effective in increasing knowledge of the roles and responsibilities of other disciplines and providing opportunities to interact with students from other health professions. Interprofessional education-trained students from multiple health-care disciplines feel comfortable performing SBIRT to identify persons at risk for substance misuse in practice.

## Introduction

Substance abuse is a significant cause of preventable death in the United States with one in four deaths attributed to complications of alcohol, tobacco, and illicit substance use (National Institute on Drug Abuse, 2012). Health-care costs associated with substance abuse are approximately $249 billion for alcohol and $193 billion for illicit drugs and given the growing opioid epidemic in America these numbers will only continue to rise (Birnbaum et al., 2011; Centers for Disease Control and Prevention, 2016; National Drug Intelligence Center, 2011). Historically, best practices in counteracting substance misuse focused on prevention and treatment interventions but did not address those at risk for substance use disorders (SUDs). A public health approach to intervene with at-risk individuals before they develop an SUD can be used to reduce the overall physical and economic costs of the disease (Aldridge, Linford, & Bray, 2017).

The comprehensive, evidence-based approach, screening, brief intervention, and referral to treatment (SBIRT), was developed to broaden interventions and identify those at risk of developing SUDs (Babor, McRee, Grimaldi, Ahmen, & Bray, 2007; Institute of Medicine, 1990). The premise behind SBIRT is to identify and intervene with those that are at moderate or high risk of health and social problems as a result of substance use. SBIRT uses validated screening tools to assess for substance use risk and incorporates motivational interviewing via *brief interventions* to encourage the desire for positive change in at-risk individuals. Best practices for all health professionals should include universal health screening for information relevant to use, abuse or dependence on alcohol, and other drugs at every patient encounter (Babor et al., 2007)

Validated screening tools include the Alcohol Use Disorders Identification Test and the Drug Abuse Screening Test-10 that screens for at-risk drug use (Babor, Higgins-Biddle, Saunders, & Moteiro, 2001; Yudko, Lozhkina, & Fouts, 2007). The tools can then be used to assess risk and determine the level of intervention. These assessment tools can be used in many different health-care settings including emergency departments, primary care clinics, urgent care, and community pharmacies.

The use of SBIRT in patients with substance misuse has been shown to be effective with a 40% reduction in alcohol use and 76% reduction in illicit drug use (Aldridge et al., 2017). Referring patients to proper treatment and promoting change to lessen high-risk behavior leading to substance abuse in its early stages, before the patient develops a full SUD, can greatly reduce health-related harm and health-care costs. High costs associated with emergency room visits and inpatient admissions due to alcohol- and drug-related use can be reduced with the use of SBIRT (Fleming et al., 2000; Estee, Wickiezer, Shah, & Mancuso, 2010). In Wisconsin Medicaid patients, an estimated cost savings of $391 per adult beneficiary was seen as a result of using low-cost outpatient services versus emergency room and inpatient services (Paltzer, Brown, & Burns, 2017). SBIRT can be used in a multitude of settings and has been shown to be effective to decrease costs; therefore, it would be imperative to provide education and training to health-care professionals in various disciplines.

Incorporating SBIRT education in undergraduate and graduate curricula for health-care disciplines has the potential to provide future health-care professionals with the education and confidence necessary to screen and perform brief interventions in those at risk for substance misuse and abuse (Guidice et al., 2015; Osborne & Benner, 2012).

The New Hampshire (NH) SBIRT Interprofessional Education (IPE) Training Collaborative, a facilitated student training program, funded through a grant from the Substance Abuse and Mental Health Services Administration under the Department of Health and Human Services, provided an opportunity to train health profession students in SBIRT along with IPE methods in doing so. The interprofessional opportunities were designed to increase the knowledge, skills, and attitudes among health profession students to engage in SBIRT as a multidisciplinary team. The objectives of the Collaborative were to permanently integrate SBIRT into course curricula at partner academic institutions, ensuring that the next generation of the health-care workforce utilizes SBIRT in clinical practice.

To guide interprofessional learning opportunities, the IPE Collaborative (IPEC) developed a framework to enhance the preparation of health profession students for working in teams. The IPEC framework uses the overarching domain of collaboration supported by four competency areas: values and ethics, roles and responsibilities, communication, and teamwork (IPEC Practice: Core Competencies, 2011; IPEC Practice:Core Competencies for Interprofessional Collaborative Practice-Update, 2016). The NH SBIRT IPE Training Collaborative Project identified several competencies to enhance the interprofessional experience of students through changes in knowledge, skills, and attitude. Competencies include as follows:

- Describe own role, responsibilities, values and scope of practice effectively to clients/patients/families and other professionals.
- Recognize and understand how one's own uniqueness, including power and hierarchy within the interprofessional team, may contribute to effective communication or interprofessional tension.
- Reflect on own values, personal and professional, and respect those of other interprofessional team members/clients/families.
- Contribute to effective interprofessional communication, including giving and receiving feedback, addressing conflict or difference of opinions, self-reflecting.

The goal of utilizing the IPEC framework is to engage individuals in adopting the competencies within their own educational institutions, promoting the vision that interprofessional collaboration contributes to better health-care outcomes (Substance Abuse and Mental Health Administration Services, 2017)

Expanding SBIRT training to multiple disciplines using IPE methods has the potential to promote the development of a team approach in clinical practice and allows multiple points of intervention in the health-care process.

## Objective

The primary objective of this study was to provide educational training sessions on SBIRT to health profession students utilizing IPE activities and assess perceptions of the training sessions and activities with regard to confidence to utilize SBIRT in at-risk patients and overall student satisfaction with SBIRT instruction.

## Methods

Students enrolled in nursing, pharmacy, social work, medicine, and physician assistant programs at five academic institutions of higher learning across the State of NH received SBIRT training (Table 1). SBIRT training occurred from February 1 to September 30, 2016. A coordinating council consisting of SAMSHA grantees from the five institutions accessed and reviewed materials provided by SAMSHA and used these materials to facilitate campus-specific trainings (Substance Abuse and Mental Health Administration Services, 2017). The overall design of the project was to ensure that students received training on SBIRT within their own discipline at their individual campuses. The training was individualized by campus. Methods of content delivery chosen by facilitators included problem-based learning using patient, self-paced online modules using videos and lecture capture technology or didactic lecture content. Each faculty member in the learning collaborative assigned activities and grading elements consistent with existing course work. The length and depth of the content were individualized, although the objectives for the trainings were applied consistently across programs. For example, the physician assistant students received instruction via classroom presentations, online simulations, and video-recorded practice.

**Table 1.** Background Demographics.

	Trained (no.)	Completed survey (%)
Discipline		
Nursing/social work	82	62
Medical	98	45
Pharmacy	77	40
Physician assistant	23	61
Mental health/counseling	23	48
Total	303	50
	Students who completed survey	Percentage
Gender^a^		
Male	40	30%
Female	94	70%
Race^b^		
Black	12	8%
Asian	19	13%
White	99	69%
Alaskan	0	0%
Native American	1	1%
Native Hawaiian	12	8%
Ethnicity^c^		
Hispanic	5	4%
Non-Hispanic	128	96%

a 17 students did not respond to gender.

b Students could select multiple races, 27 did not respond.

c 18 students did not respond to ethnicity.

The faculty facilitators used training resources, which were appropriate for integration into current curricular offerings acknowledging that students were at different phases and stages of their health profession programs. For example, first-year medical students, sophomore undergraduate nursing students, or pharmacy doctoral students completing clinical rotations. The pedagogy of the offerings was also consistent with the individual faculty teaching methods.

Following this profession-specific instruction, students were asked to apply their knowledge and skills using the SBIRT process by participating in one or both of two interprofessional activities. One activity was an asynchronous student-centered case-based learning activity. The activity was facilitated by faculty members of the learning collaborative. The discussion board feature of the online learning platform was used to facilitate interprofessional communication across schools and disciplines. Students from participating schools were randomly assigned into multidisciplinary groups and were asked to reflect on an unfolding patient case with a substance misuse-related problem, taking the perspective of not only their own profession but also that of another member of a different discipline in the group. For example, a nurse might reflect on the use of the nursing process when encountering a patient with an SUD, while a physician assistant may approach the case using diagnostic reasoning. The main goal of the learning activity was perspective-taking, which could translate to cooperative use of SBIRT in practice. The activity lasted for 3 weeks followed by student evaluation of the activity.

The second activity involved what the group called *IPE Day*. This was a face-to-face conference, which engaged students in interprofessional learning activities focusing on the use of SBIRT. Learning activities included a discussion of health-care professional's roles in SBIRT, SUD treatments, integrating SBIRT into practice, and training on the use of naloxone. IPE learning activities were guided by the IPEC Core Competencies for Interprofessional Collaborative Practice (IPEC Practice: Core Competencies, 2011; IPEC Practice:Core Competencies for Interprofessional Collaborative Practice-Update, 2016).

On completion of SBIRT training, students were given an online 24-question Likert-type survey related to knowledge, skills, and attitudes regarding SBIRT and satisfaction with the training and instruction. Similar survey formats were used for both the asynchronous learning and IPE Day activities. A Likert-type scale was chosen as a means of evaluation as the questions focused on the assessment of attitudes and beliefs and is considered best practice for public health evaluations (Sullivan & Artino, 2013). In addition, Likert-type scale survey items allow for the use of parametric tests to analyze and interpret survey results. Usefulness of the training was measured using a 4-point scale and confidence to apply SBIRT in practice was measured using a 5-point scale. Satisfaction and relevance were aggregated by the collective *strongly agree* and *agree* responses. Negative responses to questions were registered if the student answered, *disagree* or *strongly disagree*. Responses were collected by the Center for Program Design & Evaluation at Dartmouth. In addition to the survey items using a Likert-type scale, the evaluation also included open-ended questions about the most useful parts of the training and ways in which the training could be improved. Responses to open-ended survey items were analyzed using grounded theory technique in which survey respondent comments were coded according to theme and content. Coded themes that emerged were then grouped into larger categories as additional data were collected and analyzed. Although the funding agency did not require a review by an institutional review board, MCPHS University and Dartmouth Hitchcock Medical Center approved this research.

## Results

**Figure 1.**
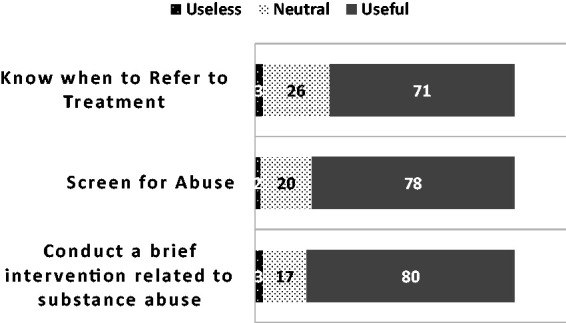
Usefulness of SBIRT training to build skills (%).

A total of 303 students completed SBIRT training and of those 50% completed the posttraining survey. The majority of students were female (70%) and White (69%; Table 1). A total of 80% of the students believed that the training was useful in teaching them to conduct brief interventions related to substance abuse, 78% reported that they had the skills to successfully screen for abuse, and 71% reported that they knew when to refer to treatment (Figure 1).

**Figure 2.**
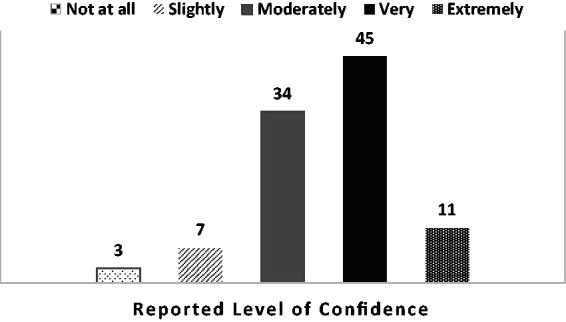
Student confidence applying SBIRT to client or patient (%).

After completing SBIRT training, 97% of students agreed that SBIRT was relevant to their future patients and clients. Confidence to apply what was taught in clinical practice with a client or patient was reported at 34% feeling moderately confident, 45% very confident, and 11% extremely confident (Figure 2).

**Figure 3.**
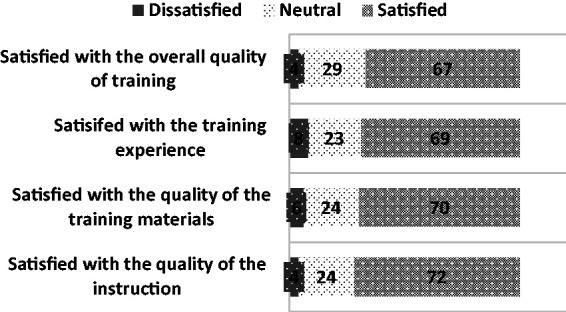
Satisfaction with SBIRT training (%).

Overall, 72% of students reported that they were satisfied with the overall quality of the SBIRT instruction at their institution. Satisfaction with the training materials was reported by 70% of the students. Student satisfaction with the overall quality of the SBIRT training was reported at 67% (Figure 3). Qualitative analysis categorized the most useful part of the training programs for the students to be the screening skills, SBIRT materials, and patient case role-play scenarios, 22%, 22%, and 15%, respectively.

Of the 303 students trained, 76 participated in the asynchronous online IPE activity of those students (*n* = 24), reported a range between 57% and 73% that the activity was *very effective* or *extremely effective* in increasing knowledge of the roles and responsibilities of other disciplines and providing opportunities to interact with students from other health professions. Twenty-five of the 32 participants who attended the face-to-face interprofessional day reported that it was *very effective* or *extremely effective* in helping them build specific IPE competencies 92% and 100%, respectively.

## Discussion

The student responses showed positive perceptions of SBIRT and confidence in applying the techniques in their future careers. The results of the study showed that multiple types of health-care providers respond positively to SBIRT training. The multidisciplinary health-care students reported increased knowledge about the subject and confidence using SBIRT in their future careers. The evaluation results were similar to studies done individually with medical residents and social workers, where students had a greater understanding of substance use and abuse after completing SBIRT training (Guidice et al., 2015; Osborne & Benner, 2012). Interestingly, our study included a variety of health profession students and academicians in several higher education institutions promoting the use of IPE and collaboration to effectively deliver SBIRT training in contrast to previous research that was done with pediatric medical residents alone (Guidice et al., 2015). The results of this study show that health-care students are uniformly receptive of SBIRT training despite difference in profession and form of delivery, similar to prior studies (Guidice et al., 2015). The favorability of SBIRT and the confidence seen in the students show the potential for SBIRT training to be included in the curriculums of many health-care professions and therefore increasing the opportunity to engage with those at risk for SUDs in practice.

Limitations of the study included the varied teaching methods, and faculty instructors used to deliver the SBIRT information to students based on the institution. Although the training materials were similar for all teaching faculty, the training delivery techniques and curricular format differed at each institution. For example, some institutions embedded the training session in existing courses, and others offered the sessions to students while on clinical rotations and utilized online methods similar to what was used in a prior study with pediatric medical residents (Guidice et al., 2015)

Another limitation of our study involved the varied student participation at each institution. At some institutions, the training was voluntary, while at others, it was mandatory and graded. The nonmandatory nature of the SBIRT training possibly contributed to the low overall survey completion rate of 49%.

The majority of students reported that they believed SBIRT training was useful at improving their skills; however, the level of confidence performing the SBIRT intervention varied where a little over 50% of the students reported that they were *very* or *extremely* confident in utilizing their SBIRT training, and 34% reported that they had *moderate* confidence using the technique. Providing more SBIRT practice using case simulations and role-play activities during the training sessions could contribute to advanced application of SBIRT and increase the confidence level of performing SBIRT for students.

## Implications for Practice

Adopting a public health model that provides a larger number of opportunities to intervene with at-risk behaviors is ideal in a patient-centered delivery of health care. A plan to achieve this goal would be to cast a wide net by increasing the number of health-care professionals trained and available to screen, intervene, and refer at-risk patients across the continuum. The use of universal screening could be used as a valuable tool in increasing the number of patients who discuss their substance use with a health-care professional. The opportunity for open communication has the potential to prevent the development of SUD as well as reducing the number of patients who are at risk for developing physical and mental health effects of SUD.

Providing training to a larger and more diverse array of health-care professionals allows patients to be screened at multiple levels of the health-care system from the intensive care unit to the community pharmacy. Like any chronic condition, such vigilance is imperative when dealing with difficult issues such as substance misuse.

Despite its limitations, the study showed a positive student response to SBIRT training, increased knowledge about the technique, and strong perceived confidence in utilizing SBIRT in their future health-care careers. Utilizing the student comments about the SBIRT training could help instructors design more efficient SBIRT training sessions and help them integrate SBIRT training into the students' curriculums.

## Conclusion

The study concludes that students from multiple health-care disciplines believe in the usefulness of SBIRT training. Increasing the number of SBIRT trained providers could help better direct at-risk patients to the proper treatments and overall reduce substance abuse. Future research to assess the application of the training by evaluating student's use of SBIRT, while on clinical rotations would provide additional knowledge regarding the effectiveness of the training.

## Authors’ Note

This research has been presented at the following venues: Primary Care 2020: Future challenges, tips for today, Harvard Medical School, Boston, MA; New England Rural Health Conference, Bartlett, NH; and Association for Medical Education and Research in Substance Abuse Conference, Washington, DC.
